# Pathway based therapeutic targets identification and development of an interactive database CampyNIBase of *Campylobacter jejuni* RM1221 through non-redundant protein dataset

**DOI:** 10.1371/journal.pone.0198170

**Published:** 2018-06-08

**Authors:** Mohammad Uzzal Hossain, Taimur Md. Omar, Iftekhar Alam, Keshob Chandra Das, A. K. M. Mohiuddin, Chaman Ara Keya, Md. Salimullah

**Affiliations:** 1 Bioinformatics Division, National Institute of Biotechnology, Ganakbari, Ashulia, Savar, Dhaka, Bangladesh; 2 Department of Biotechnology and Genetic Engineering, Life Science Faculty, Mawlana Bhashani Science and Technology University, Santosh, Tangail, Bangladesh; 3 Plant Biotechnology Division, National Institute of Biotechnology, Ganakbari, Ashulia, Savar, Dhaka, Bangladesh; 4 Molecular Biotechnology Division, National Institute of Biotechnology, Ganakbari, Ashulia, Savar, Dhaka, Bangladesh; 5 Department of Biochemistry and Microbiology, North south University, Bashundhara, Dhaka, Bangladesh; Massey University, NEW ZEALAND

## Abstract

The bacterial species *Campylobacter jejuni RM1221* (*CjR*) is the primary cause of campylobacteriosis which poses a global threat for human health. Over the years the efficacy of antibiotic treatment is becoming more fruitless due to the development of multiple drug resistant strains. Therefore, identification of new drug targets is a valuable tool for the development of new treatments for affected patients and can be obtained by targeting essential protein(s) of *CjR*. We conducted this *in silico* study in order to identify therapeutic targets by subtractive *CjR* proteome analysis. The most important proteins of the *CjR* proteome, which includes chokepoint enzymes, plasmid, virulence and antibiotic resistant proteins were annotated and subjected to subtractive analyses to filter out the *CjR* essential proteins from duplicate or human homologous proteins. Through the subtractive and characterization analysis we have identified 38 eligible therapeutic targets including 1 potential vaccine target. Also, 12 potential targets were found in interactive network, 5 targets to be dealt with FDA approved drugs and one pathway as potential pathway based drug target. In addition, a comprehensive database ‘CampyNIBase’ has also been developed. Besides the results of this study, the database is enriched with other information such as 3D models of the identified targets, experimental structures and Expressed Sequence Tag (EST) sequences. This study, including the database might be exploited for future research and the identification of effective therapeutics against campylobacteriosis. URL: (http://nib.portal.gov.bd/site/page/4516e965-8935-4129-8c3f-df95e754c562#Banner).

## Introduction

The genus *Campylobacter* is composed of a wide variety of non-spore forming Gram-negative bacteria that are predominantly rod or spiral shaped [1]. Most *Campylobacter* infections are acquired through contaminated food and two species, *Campylobacter coli* and *Campylobacter jejuni* are the primary cause of the human disease termed as campylobacteriosis [2,3]. *Campylobacter jejuni (C*. *jejuni)* is the species that induces acute gastroenteritis and bacterial food poisoning in infected patients. Normal infection with *C*. *jejuni* causes uncomplicated gastroenteritis but severe infection may result in abdominal cramps, fever or even serious diseases like diarrhea, GuillainBarré Syndrome or Miller Fischer Syndrome [4–6]. *C*. *jejuni* infection is acquired via numerous sources associated with lack of awareness such as undercooked livestock meat, poultry, unpasteurized milk or contaminated water sources [7].

*C*. *jejuni* causes the highest proportion of campylobacteriosis cases in developed countries, and in the United States, between $1.3 to $6.8 billion dollars is spent annually for treating the illness [8,9]. According to the ECDC (European Centre for Disease Prevention and Control) and EFSA (European Food Safety Authorities) report the most important zoonosis was found to be campylobacteriosis compared to yersinosis and salmonellosis [10–12]. Global campylobacteriosis incidence is increasing each year and has almost exceeded the incidence of shigella infections [13,14]. In 2010 New Zealand endured one of the highest rates of campylobacteriosis demonstrating that *Campylobacter* infection is a global threat [10].

*CjR* is a disease causing strain which has similar *C*. *jejuni* type of infectious properties. Antibiotic treatment against *CjR* is becoming increasingly more ineffective due to the emergence of multiple antibiotic resistance strains. This resistance requires special attention as *C*. *jejuni* is capable of efficient transfer of the resistant genes into other strains. In 2000, data of primary genome sequence of *C*. *jejuni* was released but detailed information about variations and polymorphisms in the complete genome sequences of different strains was only published in 2006 [15–17]. Currently, information about gene/protein sequences and metabolic pathways of *CjR* is available in various databases like NCBI, KEGG, Biocyc.org etc. These databases have become a critical tool for the discovery and identification of new molecular target(s) and subsequently provide a valuable platform for researchers and the pharmaceutical industry to enable the development of new drugs and vaccines. Targets should normally be an eligible gene or protein of a specific strain which can be targetable by an existing or non-existing drug.

Conventional drug target identification is time consuming, expensive, laborious and often only a few drug targets can be identified. In comparison, the *in silico* approach allows for a great deal of analysis to be carried out within a short period of time which is cost effective and often delivers a high number of the promising drug targets from a large pool of information. This has been facilitated by information available from various databases that provide whole genome sequences of various organisms ranging from pathogenic bacteria to human [18,19]. Currently, utilization of various *in silico* approaches to identify potential vaccine or drug targets has become a prerequisite for drug and vaccine design [20–22]. One such *in silico* approach is Subtractive genomic analysis which attempts to discover new proteins or targets that are important for the survival of the pathogenic microorganism and non-homologous to the human host. Designing drugs against these proteins have a high probability to be effective against their target microorganism [23]. In this study, we applied this subtractive proteome analysis to identify essential proteins such as chokepoint enzymes, virulent proteins and antibiotic resistant proteins that are important for the survival of *CjR*. This Subtractive analysis is possible due to the genome of *CjR* becoming available in various web based databanks. After identification and confirmation of human non-homologues drug targets are characterized to facilitate effective drug design. Additionally, we have also analyzed and suggested pathways for future drug targets and created an open access database named CampyNIBase to store all identified and characterized therapeutic targets. Furthermore, in order to enrich the database, other relevant campylobacter information such as expressed sequence tags (EST) sequences, experimental drugs are incorporated in CampyNIBase through extensive surveying of the literature.

## Materials and methods

**Fig 1:**
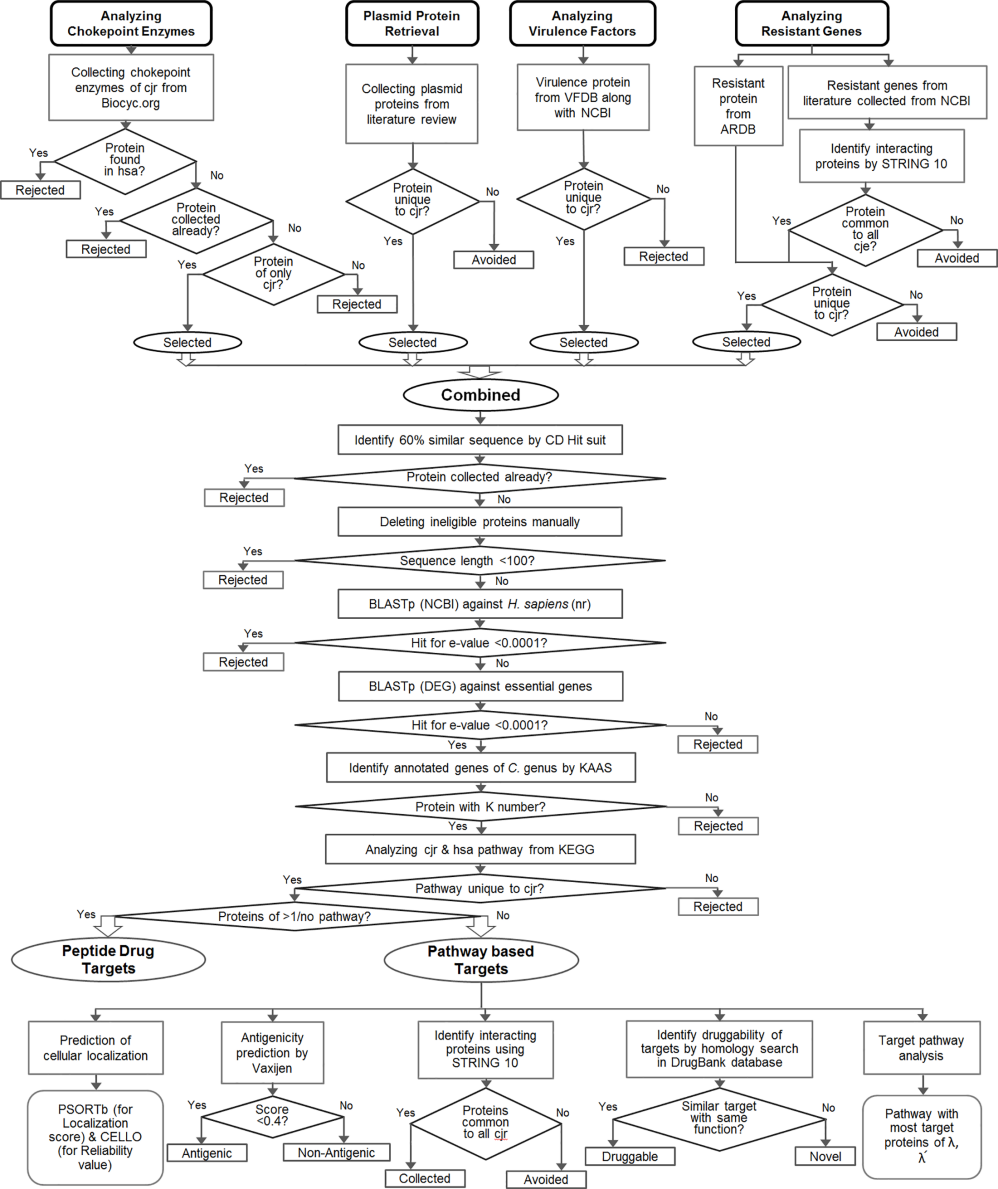
Complete flowchart of whole subtractive analysis. The workflow is represented as flowchart showing subtractive analysis of the proteins in each step.

The identification of drug targets was carried out in three phases. In phase-I, major proteins of *CjR* genome were collected from different sources. These proteins include chokepoint enzymes, plasmid proteins, virulent proteins and antibiotic resistant proteins. In phase-II, subtractive analyses were carried out through different steps by excluding human homologues proteins and collecting important proteins required for the survival of *CjR*. In phase-III, potential drug targets found from subtractive analyses in phase-II are characterized. The entire work flow can be seen as a flowchart (Fig 1).

### Phase–I: Collection of *CjR* proteins

#### Analyzing chokepoint enzymes

**Fig 2:**
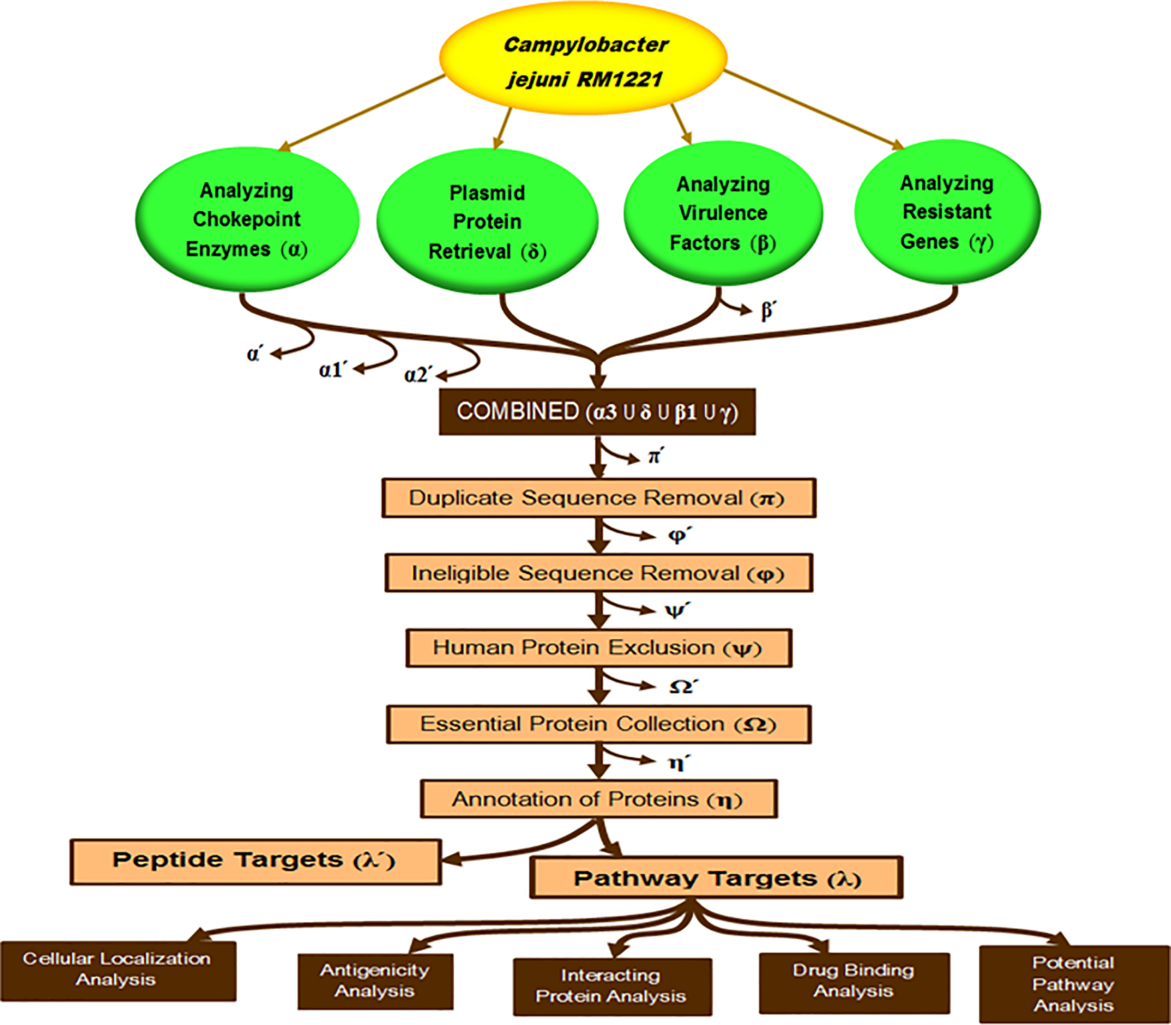
Outline of subtractive analysis and list of proteins. The enlisted proteins were prioritized (different symbol) from different phase of subtractive channel of analysis.

Chokepoint enzymes are available in Biocyc (Biocyc.org) [24], a server containing a collection of Pathway/Genome Databases (PGDBs) of various organisms. In the database server, *CjR* was selected as desired. Then each of the enzymes involved in chokepoint reactions on the consuming side and producing side was collected. In this stage, only EC numbers and names of the enzymes were collected. By the same process, those of human chokepoint enzymes were also collected. This is a vital step as proteins with similar domains to a human enzyme may lead to harmful drug or vaccine side effects in treated patients. Subsequently enzymes of *CjR* similar to proteins expressed by humans were excluded to avoid cross targeting. Next the EC numbers or names collected more than once were rejected to reduce manual work. Finally, FASTA sequences were obtained and only the enzymes unique to the *CjR* strain were collected. A list of the selected enzymes was collected as (α3) list (Fig 2).

#### Plasmid protein retrieval

Plasmids of *C*. *jejuni* can contain unique characteristics compared to other *Campylobacter* species. Plasmid sequences were retrieved as those characteristics can also be potential drug targets. The protein sequences were retrieved from literature review [25]. Only the unique proteins of *CjR* were collected and listed as delta (δ) list (Fig 2).

#### Analyzing virulence factor

Virulence factors expressed by bacteria are required for the survival and success of pathogenic bacteria and are potential drug targets. They were identified and collected from the virulence factor database (VFDB) [26]. To collect all known virulence factors of *CjR*, the NCBI database [27] linked with VFDB was useful. All the retrieved proteins were listed as beta (β) list: Proteins unique to *CjR1* strain were selected in (β1) list and other proteins were excluded and listed in (β1’) list (Fig 2).

#### Analyzing antibiotic resistant genes

Many of the strains of *C*. *jejuni* have developed resistance to multiple drugs. Genetic mutations in certain genes of *C*. *jejuni* are responsible for such kind of resistance [28–30]. The proteins interacting with these resistant gene components were identified by web based tool, Search Tool for the Retrieval of Interacting Genes (STRING 10) [31]. Common proteins found in all *C*. *jejuni* strains that were available in the STRING 10 database and known for interacting with resistant genes were collected in Excel file. The FASTA sequences of these proteins were retrieved from NCBI protein database [27] and some additional resistant proteins were retrieved from Antibiotic Resistance Genes Database (ARDB) [32]. The FASTA sequence of all of the proteins was collected and listed as gamma (γ) list (Fig 2). Only the proteins unique to *CjR* were collected and others were avoided (Fig 2).

### Phase–II: Subtractive analysis of drug targets

#### Duplicate sequences removal

As the FASTA sequences of the proteins were collected from different sources, many of these were collected more than once. Thus a sequence clustering web server, CD-HIT Suite [33] was used to compare all the retrieved sequences and remove duplicates. The input was 60% in sequence identity as the cut-off parameter and selected proteins were collected in (π) list (Fig 2).

#### Ineligible sequence removal

Proteins with sequence length less than 100 were considered ineligible, removed manually and listed in (φ´) list, whereas proteins with length ≥ 100 were enlisted in (φ) list (Fig 2).

#### Human protein exclusion

If the target protein of *CjR* is homologous to a human protein, then the drug or vaccine developed against it has potential to also target the human protein. Hence, proteins homologous to human proteins were removed. For this purpose, proteins of (φ) list were submitted to BLASTp [34] against a non-redundant database of *Homo sapiens*. The considered threshold was 10^−3^. The proteins non-homologous to human were collected and listed as (ψ) list (Fig 2).

#### Essential protein collection

*C*. *jejuni* is one of the leading causes of food poisoning and diarrhea [4–6]. Therefore, other strains responsible for food poisoning and diarrhea were selected for essential protein analysis. The major bacteria responsible for food poisoning are *Clostridium perfringens*[35], *Salmonellaspp*. [36], *Escherichia coliO157*:*H7* [37], *Bacillus cereus*[38], *Listeria monocytogenes*[39], *Shigella spp*. [40], *Staphylococcus aureus*[41], *Staphylococcal enteritis*[42], *Streptococcus*[43,44], *Vibrio cholerae*[45], *Vibrio parahaemolyticus*[46], *Vibrio vulnificus*[47], *Yersinia* enterocolitica [48] *Brucella spp*[49], *Coxiella*burnetii [50] and *Plesiomonas* shigelloides [51]. *Escherichia coli, Salmonella, Shigella*, *Clostridium* difficile [52], *Staphylococcus aureus*[53] can cause diarrhea. A BLAST search was performed for proteins of (ψ) list against the essential proteins of common organisms mentioned above from the DEG 10 [54] database. BLASTp program analysis was performed in Protein Query vs. Protein Database. The expect value was taken as 1E-10 and essential proteins were enlisted in (Ω) list (Fig 2).

#### Annotation of proteins

In the Kyoto Encyclopedia of Genes and Genomes (KEGG) database [55], genes in complete genomes are annotated with the KEGG orthology (KO) identifiers, or the K numbers. The essence of the KO system is that it is a pathway based definition of orthologous genes. The KO entry represents an ortholog group that is linked to a box (gene product) in the KEGG pathway diagram. Thus, once the KO identifiers, or the K numbers are assigned to genes in the genome, which is manually verified in KEGG, organism-specific pathways can be computationally generated. Proteins of (Ω) list were subjected to BLASTp against 10 strains of “Campylobacter” found in KEGG Automatic Annotation Server (KAAS) [56]. Proteins with KO number were collected and others were excluded. Selected proteins were enlisted in (η) list with KO numbers (Fig 2).

#### Pathway analysis

Pathways of the *CjR* proteins were revealed from the KEGG [55] database. If a protein of *C*. *jejuni*is involved in the same pathway found in humans then the drug target can be harmful for the host. Therefore proteins common to *C*. *jejuni* and *H*. *sapiens* pathway were excluded to avoid cross targeting and only proteins unique to pathways in *C*. *jejuni* were collected. To find out pathway based drug targets, proteins involved in only one pathway were collected and enlisted in (λ) list (Fig 2).

### Phase–III: Characterization of pathway based drug targets

A pathway based drug is a drug which targets whole pathway rather than single protein. Proteins known to be involved in one pathway were considered as major targets as targeting one pathway is easier to produce a pathway based drug. These are the proteins enlisted in lambda (λ) list and several characteristic features of these proteins were figured out.

#### Subcellular location analysis

Proteins expressed on the surface of bacteria are probable vaccine antigen candidates while proteins found in the inner membrane regions and cytoplasm of the bacteria are probable drug candidates. Thus, knowing the locations of the proteins is extremely useful and necessary. A bacterial protein subcellular localization (SCL) predictor, PSORTb 3.0 [57], and CELLO v.2.5 [58], a server based on a two-level support vector machine (SVM) system predicting subcellular localization of the proteins were used. As a protein’s function is related to its localization, this prediction would be used for functional analysis.

#### Antigenicity analysis

To predict protective antigens of bacteria, Vaxijen [59], an alignment-independent prediction server, was used. It reveals the antigenic score of every protein. The proteins with higher antigenic score were considered as a more viable antigen target. Proteins with antigenic score less than 0.4 (default threshold value) were considered as non-antigenic proteins. We used NetCTL 1.2 server (http://www.cbs.dtu.dk/services/NetCTL/) and AllerTOP v.2.0 server (http://www.ddg-pharmfac.net/AllerTOP/) for the identification of potential T- cell epitopes from the proteins that had high antigenic scores. Immune Epitope Database (IEDB) [60] tools were utilized for MHC-I molecules interactions with potential T- cell epitopes as well as epitope conservancy analysis. Further, we employed a set of bioinformatics tools including Kolaskar and Tongaonkar antigenicity scale [61], Emini surface accessibility prediction [62], Karplus and Schulz flexibility prediction [63], Bepipred linear epitope prediction analysis [64] and Chou and Fasman beta turn prediction analysis [65, 66] to predict the B-cell antigenicity.

#### Interacting protein analysis

A protein functions in a system of an organism by interacting with other proteins. To characterize such interacting proteins of each target of (λ) list STRING 10 [31], a web based search tool, was used. As this tool does not have information about *CjR* strain, we collected common interacting genes among available *C*. *jejuni* strains for each target. The target with a greater number of edges, interacts with more genes (nodes) and has a higher importance in a system. If a target forms numerous interactions with other proteins, it might be termed as ‘hub’ [31].

#### Drug binding analysis

Another BLASTp program was setup to know whether targets can be treated by current Food and Drug Administration (FDA) approved drugs. So, each target in λ list were subjected to BLASTp against DrugBank 3.0 target collection [67] for homology comparisons. The hit targets from λ list with DrugBank can be dealt with approved and available drugs.These targets were named as ‘Druggable’ targets. Targets nonhomologous to targets of the DrugBank 3.0 target collection were termed as ‘Novel’ targets. Furtheremore, Autodock Vina [68] was utilized to perform the blind docking to predict the binding affinity between druggable targets and predicted drug molecules.

#### Potential pathway analysis

Drugs can be designed to target whole pathways rather than a single protein. So, a pathway containing more targets of η list was considered as a pathway with greater potential. Here, we only took pathways of λ list for manual analysis. Pathways containing proteins involved in human pathways were ignored. Pathways containing the highest number of proteins of η list were considered as the most potential pathway.

#### Experimental and tertiary structure identification

The PDB [69] database was scanned to identify the experimental structures with the query of identified sequences of therapeutic targets. The available structures were deposited in our database ‘CampyNIBase’ whilst the remaining sequences that showed no hit to the PDB database were also employed to build 3D structures for facilitating the drug discovery. We have selected the best template from the Local meta-threading-server (LOMETS) [70] where more than one threading program showed the same template. Modeller 9.17 [71] was used for generating a number of models. Subsequently, DOPE (Discrete Optimized Protein Energy) scoring was considered to select the best model from a number of generated models. Thereafter, quality assessment of built models was checked by Ramachandran Plots [72]. We also employed the COFACTOR server [73] for the prediction of binding site in the generated models.

#### Database development and organization

We have developed a user friendly open access database named CampyNIBase in which all the identified drug targets were deposited. The storage system for this database was based on My-SQL hosted by Bangladesh Computer Council Data Centre. The user interface and back-end of this database was based on an open source scripting language PHP. The contents of our developed database were categorized with different types of menu bar.

## Results

### Annotation of *CjR* proteome

Data retrieved from the Biocyc database revealed that *CjR* contains 238 and 251 chokepoint reactions on the consuming and producing side respectively [24]. Among them, 236 chokepoint enzymes were involved in consuming chokepoint reactions and 260 chokepoint enzymes were involved in producing chokepoint reactions. Thus, the number of total consuming and producing enzymes is 496 collected in alpha (α) list. After excluding 195 human enzymes (α´ list), 301 chokepoint enzymes remained (α1 list). 107 enzymes (α1´ list) were found to be collected more than once and after their removal, 194 enzymes (α2 list) remained. While collecting the FASTA sequences, it was found that all of these enzymes were not unique to the *CjR* strain. Therefore following the exclusion of 91 proteins (α2´ list) of other strains, 103 chokepoint enzymes (α3 list) were selected to remain (Table 1, S1 Table). Two plasmid proteins of *Campylobacter* were found from literature review [25]. Among them one was specific for *CjR*. This protein was enlisted as delta (δ) list (Table 1, S2 Table). A total of 2204 virulence proteins were collected from VFDB [26] along with NCBI database [27] and assigned to beta (β) list. Among them 104 proteins were manually identified as common to other strains of *C*. *jejuni* and collected in (β´) list followed by rejection. Therefore, 2100 proteins unique to *CjR* were collected and enlisted in (β1) list (Table 1, S3 Table). From literature reviews, the cmeB [28],gyrA [29] and *aphA-*7 [30] antibiotic resistant genes were collected. In *C*. *jejuni*, *cmeB* and *gyrA* were found to interact directly with 1 and 5 proteins of *CjR* respectively but *aphA-7* did not interact with other proteins. In this step, a total of 9 proteins were found as the number of resistant proteins along with interacting proteins. Also from VFDB [26], 5 proteins were retrieved. So, the number of total proteins from this analysis is 14 and they were listed in gamma (γ) list (Table 1, S4 Table). All of the chokepoint enzymes, plasmid proteins, virulent proteins and antibiotic resistant proteins listed in α3, δ, β1 and γ lists respectively were combined (S5 Table) to obtain a total of 2218 proteins for subtractive analyses.

**Table 1:** List of targets through identification channel.

**Phase**	**Analysis**	**Process**	**Collected for Analysis**	**Rejected**	**Selected**	**Final selected proteins from a phase**
**I**	Analyzing chokepoint enzymes	(i) Rejecting proteins of hsa	**496**(α)	**195**(α´)	**301**(α1)	**2218**
		(ii) Rejecting duplicate proteins	**301**(α1)	**107**(α1´)	**194**(α2)	
		(iii) Selecting unique proteins of cjr	**194**(α2)	**91**(α2´)	**103**(α3)	
	Plasmid protein retrieval	Collecting protein from literature review	**1**(δ)	**0**	**1**(δ)	
	Analyzing virulence factor	Selecting unique proteins of cjr	**2204**(β)	**104**(β´)	**2100**(β1)	
	Analyzing antibiotic resistant genes	Selecting unique proteins of cjr	**14**(γ)	**0**	**14**(γ)	
**II**	Duplicate sequence removal	60% sequence identity cut-off	**2218**	**407**(π´)	**1811**(π)	**38**
	Ineligible protein removal	Removing sequence length <100	**1811**	**240**(φ´)	**1571**(φ)	
	Human protein exclusion	Removing proteins homologous to Human by BLASTp	**1571**	**384**(ψ´)	**1187**(ψ)	
	Essential protein collection	Collecting essential proteins by BLASTp against DEG database	**1187**	**802**(Ω´)	**385**(Ω)	
	Annotation of proteins	Proteins with KO number collection from KAAS	**385**	67(η´)	**318**(η)	
	Pathway analysis	Identify unique pathway and proteins of 1 pathway from KEGG	**318**	**280**(λ´)	**38**(λ)	
**III**	Subcellular Location Analysis	PSORTb and CELLO protein location	38(λ)			
	Antigenicity Analysis	Vaxijen score for antigenicity	**38**(λ)			
	Broad Spectrum Analysis	Interacting protein analysis	**38**(λ)			
	Druggability Analysis	Targets in Drug Bank	**38**(λ)			

### Identification of potential therapeutic targets

#### Recognition of human non-homologous protein of *CjR*

At the beginning of therapeutic target identification, we have removed 407 duplicate protein sequences from the 2218 proteins obtained during the annotation of the *CjR* proteins. The duplicate sequences are listed in (π´) list. The remaining 1811 sequences listed in (π) list contains 101 chokepoint enzymes, 1 plasmid protein, 1704 virulent proteins and 5 antibiotic resistant proteins (Table 1, S6 Table). Next we manually excluded 240 (φ´) proteins with length <100 amino acids from (π) list and 1571 proteins were collected and listed in (φ) list. There are 101 chokepoint enzymes, 1465 virulent proteins and 5 antibiotic resistant proteins in (φ) list (Table 1, S7 Table). Finally, through BLASTp program [34], 1187 proteins were found to be non-homologous to proteins expressed by humans. Those proteins were retrieved and and listed (ψ). The ψ list contains 75 chokepoint,1108 virulent and 4 antibiotic resistant proteins. The remaining 384 proteins were excluded and listed as (ψ´) list (Table 1, S8 Table).

#### *CjR* specific essential proteins

Essential proteins are proteins that are required for the survival of a species in any condition. Therefore, we attempted to identify *CjR* specific essential proteins. Through DEG 10 database [54] 16 bacterial species were found to cause food poisoning and diarrhea. They are *Bacillus subtilis 168*, *Campylobacter jejuni NCTC 11168*, *Escherichia coli MG1655 I*, *Escherichia coli MG1655 II*, *Salmonella entericaserovar Typhi*, *Pseudomonas aeruginosa PAO1*, *Salmonella entericaserovar Typhimurium SL1344*, *Salmonella entericaserovar Typhimurium 14028S*, *Salmonella typhimurium LT2*, *Staphylococcus aureus N315*, *Staphylococcus aureus NCTC 8325*, *Streptococcus pneumonia*, *Streptococcus pyogenes MGAS5448*, *Streptococcus pyogenes NZ131*, *Streptococcus sanguinis* and *Vibrio cholerae N16961*. After protein BLAST against DEG 10 database 385 proteins were found as essential proteins for CjR and contained 45 chokepoint proteins, 338 virulent factors and 2 antibiotic resistant proteins which are listed in (Ω) list. The remaining 802 proteins were rejected and listed in (Ω´) list (Table 1, S9 Table).

#### Pathway based identification of therapeutic targets

385 *CjR* specific essential proteins were processed with KEGG Automatic Annotated Server (KAAS). Following the processing procedure, 318 proteins were found in KEGG Ortholog (KO) list with K numbers and collected in (η) list containing 45 chokepoint proteins, 271 virulence proteins and 2 antibiotic resistant proteins. The other 67 proteins were removed and listed as (η´) list (Table 1, S10 Table).Using the KEGG^55^ database, 26 proteins of (η) list were found to be involved in human pathways and were removed. 38 proteins were found in only one pathway and selected as potential therapeutic targets (λ) list. Other proteins were enlisted in (λ´) list (Table 2, S11 and S12 Tables).

**Table 2:** List of pathway based drug targets.

No.	KEGG ID	Protein Name	KO Number	Pathway	Pathway ID	NCBI Protein ID	Length (a.a.)	Chokepoint Enzyme	Virulence Factor
**1**	CJE1596	holo-(acyl-carrier-protein) synthase	K00997	Pantothenate and CoA biosynthesis	cjr00770	AAW36029	115	Yes	No
**2**	CJE1405	N-acetylmuramoyl-L-alanine amidase	K01448	Cationic antimicrobial peptide resistance	cjr01503	AAW35724	659	Yes	No
**3**	CJE1810	DNA primase	K02316	DNA replication	cjr03030	AAW36232	605	No	Yes
**4**	CJE1654	ATP-dependent DNA helicase, UvrD/REP family	K03582	Homologous recombination	cjr03440	AAW36087	921	No	Yes
**5**	CJE1566	non-canonical purine NTP pyrophosphatase, RdgB/HAM1 family	K02428	Purine metabolism	cjr00230	AAW36001	200	No	Yes
**6**	CJE1554	Holliday junction DNA helicase RuvB	K03551	Homologous recombination	cjr03440	AAW35989	335	No	Yes
**7**	CJE1486	aminotransferase, DegT/DnrJ/EryC1/StrS family	K15895	Amino sugar and nucleotide sugar metabolism	cjr00520	AAW35927	376	No	Yes
**8**	CJE0667	replicative DNA helicase	K02314	DNA replication	cjr03030	AAW35822	458	No	Yes
**9**	CJE0684	(di)nucleoside polyphosphate hydrolase	K08311	RNA degradation	cjr03018	AAW35806	156	No	Yes
**10**	CJE1406	oxidoreductase, 2-nitropropane dioxygenase family	K00459	Nitrogen metabolism	cjr00910	AAW35725	363	No	Yes
**11**	CJE1401	quinone-reactive Ni/Fe hydrogenase, cytochrome b subunit	K03620	Two-component system	cjr02020	AAW35720	238	No	Yes
**12**	CJE1382	excinuclease ABC, C subunit	K03703	Nucleotide excision repair	cjr03420	AAW35702	600	No	Yes
**13**	CJE1343	HD/HDIG/KH domain protein	K18682	RNA degradation	cjr03018	AAW35664	517	No	Yes
**14**	CJE1292	transcription termination factor Rho	K03628	RNA degradation	cjr03018	AAW35614	432	No	Yes
**15**	CJE1264	general glycosylation pathway protein	K15910	Amino sugar and nucleotide sugar metabolism	cjr00520	AAW35586	386	No	Yes
**16**	CJE1228	transcription-repair coupling factor	K03723	Nucleotide excision repair	cjr03420	AAW35550	978	No	Yes
**17**	CJE1196	DNA mismatch repair protein	K07456	Mismatch repair	cjr03430	AAW35521	735	No	Yes
**18**	CJE1067	hippurate hydrolase	K01451	Phenylalanine metabolism	cjr00360	AAW35395	383	No	Yes
**19**	CJE0993	thioesterase family protein	K10806	Biosynthesis of unsaturated fatty acids	cjr01040	AAW35326	137	No	Yes
**20**	CJE0966	flagellin family protein	K02397	Flagellar assembly	cjr02040	AAW35299	750	No	Yes
**21**	CJE0951	thiol:disulfide interchange protein DsbA	K03673	Cationic antimicrobial peptide (CAMP) resistance	cjr01503	AAW35288	220	No	Yes
**22**	CJE0949	para-aminobenzoate synthase glutamine amidotransferase, component I	K03342	Folate biosynthesis	cjr00790	AAW35286	594	No	Yes
**23**	CJE0943	signal peptidase I	K03100	Protein export	cjr03060	AAW35280	282	No	Yes
**24**	CJE0892	integral membrane protein MviN	K03980	Peptidoglycan biosynthesis	cjr00550	AAW35229	483	No	Yes
**25**	CJE0890	Holliday junction DNA helicase RuvA	K03550	Homologous recombination	cjr03440	AAW35227	183	No	Yes
**26**	CJE0860	flagellar basal-body P-ring formation protein FlgA, putative	K02386	Flagellar assembly	cjr02040	AAW35197	220	No	Yes
**27**	CJE0514	ATP-dependent DNA helicase RecG	K03655	Homologous recombination	cjr03440	AAW35101	607	No	Yes
**28**	CJE0410	lipoprotein signal peptidase	K03101	Protein export	cjr03060	AAW34999	156	No	Yes
**29**	CJE0402	phosphatase, Ppx/GppA family	K01524	Purine metabolism	cjr00230	AAW34991	486	No	Yes
**30**	CJE0390	excinuclease ABC, A subunit	K03701	Nucleotide excision repair	cjr03420	AAW34979	940	No	Yes
**31**	CJE0288	carbonic anhydrase	K01673	Nitrogen metabolism	cjr00910	AAW34880	211	No	Yes
**32**	CJE0782	primosomal protein N'	K04066	Homologous recombination	cjr03440	AAW34570	617	No	Yes
**33**	CJE0771	RNA polymerase sigma-54 factor	K03092	Two-component system	cjr02020	AAW34562	416	No	Yes
**34**	CJE1897	crossover junction endodeoxyribonucleaseRuvC	K01159	Homologous recombination	cjr03440	AAW34497	160	No	Yes
**35**	CJE1845	RecA	K03553	Homologous recombination	cjr03440	AAW36267	343	No	Yes
**36**	CJE0001	chromosomal replication initiator protein DnaA	K02313	Two-component system	cjr02020	AAW34498	440	No	Yes
**37**	CJE0198	undecaprenol kinase, putative	K06153	Peptidoglycan biosynthesis	cjr00550	AAW34792	267	No	Yes
**38**	CJE0406	Conserved hypothetical ProteinTIGR00023	K08591	Glycerolipid metabolism	cjr00561	AAW34995.1	202	No	Yes

### Characterization of vaccine and drug targets

**Fig 3:**
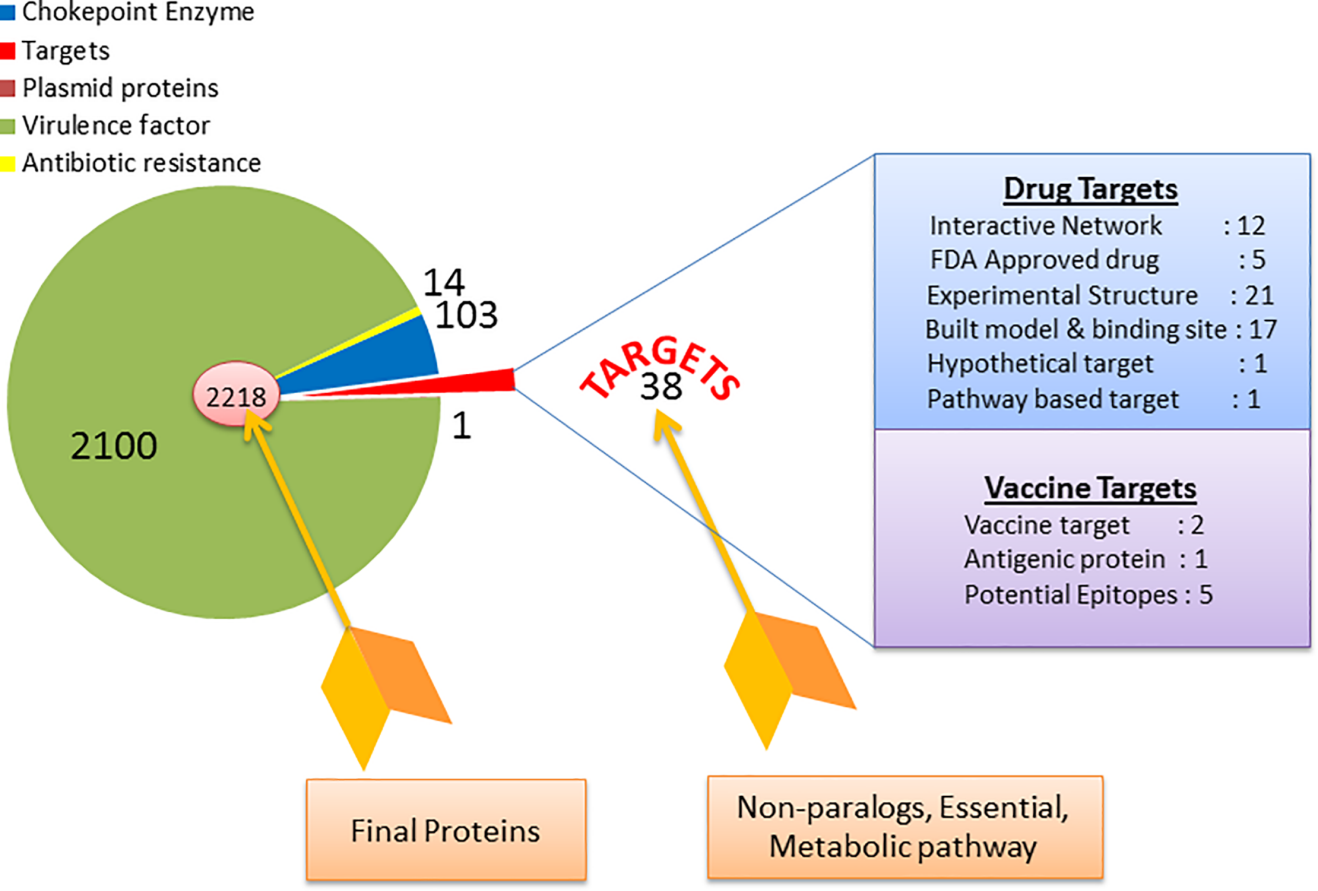
Prioritization of identified therapeutic targets. The nature of targets was categorized on the basis of their properties.

Potential target proteins were characterized for various properties such as subcellular localization, antigenicity, functional importance, druggability and involvement in pathways having more than one target protein (Fig 3). Among the 38 targets, 28 are cytoplasmic proteins, 5 inner membrane proteins, 1 periplasmic protein and 1 target has potential to be both cytoplasmic and outer membrane bound. From this result, it is obvious that 2 outer membrane proteins (1 outer membrane and 1 extracellular) are candidate vaccine targets and the other 36 targets may be potential drug targets (Table 3, S13 Table). Utilizing parameters mentioned in the method section, 19 proteins were found as antigenic proteins and the other 19 proteins were considered as non-antigenic. Among the antigenic proteins Flagellin family protein, Lipoprotein signal peptidase, ‘Quinone-reactive Ni/Fe hydrogenase, cytochrome b subunit’, N-acetylmuramoyl-L-alanine amidase and RecA were considered as the best 5 potential antigens with antigenic scores of 0.6974, 0.6913, 0.6486, 0.6402 and 0.6336 respectively (S14 Table). Among these targets the outer membrane protein N-acetylmuramoyl-L-alanine amidase, KEGG ID: CJE1405, was found with high antigenic score of 0.6402 as well as human non-allergen. Potential epitopes were searched within this protein for peptide based vaccine design approaches. Five (5) potential T-cell epitopes (SSKTLNTNY, IFVFLVFAF, LYTRSSDKF, QKFRYVVSF and DYRLVISQF) were predicted from this antigenic protein as these 9 mer amino acids showed the highest combined score of 12 Major Histocompatibility Comeplex (MHC) supertypes and human non-allergenecity (S15 Table). Therefore, this antigen has high potential to be a viable and effective vaccine target (Table 3, S14 Table). Then we have predicted the MHC-I interacting molecules on the basis of their percentile rank (>10) (S16 and S17 Tables) as the lower percentile rank is considered the strong binding affinity to T-cell epitopes[60]. The epitope conservancy analysis confirmed that these T-cell epitopes are well conserved among the CjR targets (S17 Table). We have also explored the B-cell antigenic properties from 6 different B-cell epitope prediction tools [61–66]. We have identified that the peptide region (302–310) ‘NNEKENQKP’ might be predicted as B-cell epitope from the all cross-referencing data (S1 Fig). The functional importance of each target of λ (38 nos) list was investigated. λ assuming that the more interactions a protein forms with other proteins, then the importance of that protein in a protein system also increases. Common interactions of a protein with other specific proteins among all of the strains of a specific species indicate an evolutionary importance for those interactions. For the species of *C*. *jejuni*, 12 targets available in STRING 10^31^. CJE1810, CJE1554, CJE0667, CJE1382, CJE1264, CJE0890, CJE0410, CJE0390, CJE0771, CJE1897, CJE1845 and CJE0001 showed interactions with other proteins and these interactions are common to all strains of *C*. *jejuni*. Among them CJE0890 interacts with four genes ruvB, ruvC, recN, uvrB and it has highest number of interactions. So, CJE0890 can be considered as a superior potential drug target (Table 3, S18 Table). In the current approach, the druggability of the short-listed (38 nos) potential targets was evaluated by a sequence similarity search against targets from DrugBank. Five proteins CJE1596, CJE1554, CJE1486, CJE0993 and CJE0390 were found to show affinity with FDA approved drugs as they have homology with the related proteins (Table 3, S19 Table). Among them CJE0390 is the most eligible druggable target as it interacts with more proteins (Table 3) demonstrating its importance for *CjR*. *CJE0390* is targetable by Colchicine, Silodosin, Etravirine, Doxorubicin, Gramicidin D, Cyclosporine, Adenosine triphosphate, Pravastatin, Fluvoxamine, Fluconazole, Erythromycin, Caffeine and Reserpine as inducer, substrate or inhibitor (S19 Table). The new drugs (synthesize or designed) could emerge by targeting the remaining targets ‘novel drug targets’ based on drug binding affinity, toxicity measurement and ADME (Absorption, Distribution, Metabolism and Excretion) properties.

**Table 3:** Characteristic features of pathway based drug targets.

No.	KEGG ID	Subcellular Location Analysis	Antigenicity Analysis	Common Interacting Gene	Druggability
**1**	CJE1596	Cytoplasmic	0.4297		Druggable
**2**	CJE1405	Outer Membrane	0.6402		Novel
**3**	CJE1810	Cytoplasmic	0.2813	2	Novel
**4**	CJE1654	Cytoplasmic	0.3066		Novel
**5**	CJE1566	Cytoplasmic	0.4355		Novel
**6**	CJE1554	Cytoplasmic	0.4604	2	Druggable
**7**	CJE1486	Cytoplasmic	0.4548		Druggable
**8**	CJE0667	Cytoplasmic	0.3497	1	Novel
**9**	CJE0684	Cytoplasmic	0.3053		Novel
**10**	CJE1406	Cytoplasmic	0.392		Novel
**11**	CJE1401	Inner Membrane	0.6486		Novel
**12**	CJE1382	Cytoplasmic	0.3991	2	Novel
**13**	CJE1343	Cytoplasmic	0.4653		Novel
**14**	CJE1292	Cytoplasmic	0.3294		Novel
**15**	CJE1264	Cytoplasmic	0.2672	1	Novel
**16**	CJE1228	Cytoplasmic	0.3231		Novel
**17**	CJE1196	Cytoplasmic	0.3961		Novel
**18**	CJE1067	Cytoplasmic	0.4554		Novel
**19**	CJE0993	Cytoplasmic, Outer Membrane	0.3742		Druggable
**20**	CJE0966	Extracellular	0.6974		Novel
**21**	CJE0951	Periplasmic	0.3157		Novel
**22**	CJE0949	Cytoplasmic	0.2475		Novel
**23**	CJE0943	Cytoplasmic Membrane	0.4205		Novel
**24**	CJE0892	Inner Membrane	0.5468		Novel
**25**	CJE0890	Cytoplasmic	0.422	4	Novel
**26**	CJE0860	Cytoplasmic	0.4081		Novel
**27**	CJE0514	Cytoplasmic	0.3515		Novel
**28**	CJE0410	Inner Membrane	0.6913	2	Novel
**29**	CJE0402	Cytoplasmic	0.2989		Novel
**30**	CJE0390	Cytoplasmic	0.4635	3	Druggable
**31**	CJE0288	Cytoplasmic	0.2426		Novel
**32**	CJE0782	Cytoplasmic	0.3231		Novel
**33**	CJE0771	Cytoplasmic	0.3234	1	Novel
**34**	CJE1897	Cytoplasmic	0.5022	3	Novel
**35**	CJE1845	Cytoplasmic	0.6336	2	Novel
**36**	CJE0001	Cytoplasmic	0.439	3	Novel
**37**	CJE0198	Inner Membrane	0.3805		Novel
**38**	CJE0406	Inner Membrane	0.586		Novel

Finally, to design drugs against a pathway, multiple potential pathways were analyzed. Pathways having more target proteins were considered as more potential target pathways. For this, 17 pathways were analyzed. Among them 6 pathways were found to have proteins connected to functions in human pathways and were excluded. Among the remaining 11 pathways, peptidoglycan biosynthesis contains 14 targets, the highest number of target proteins compared to other pathways. Therefore, peptidoglycan biosynthesis was considered as the pathway with most potential for discovering a suitable drug target (S20 and S12 Tables).

#### Database development

We have also exploited the experimental and 3D structure of our identified 38 drug targets. We have found 21 experimental structures from the available database. We then built 17 3D structures from the remaining sets of targets. The quality of predicted models was satisfactory, however some of the models showed the favored residues below 90%. Binding sites was also predicted by utilizing Cofactor server.

**Fig 4:**
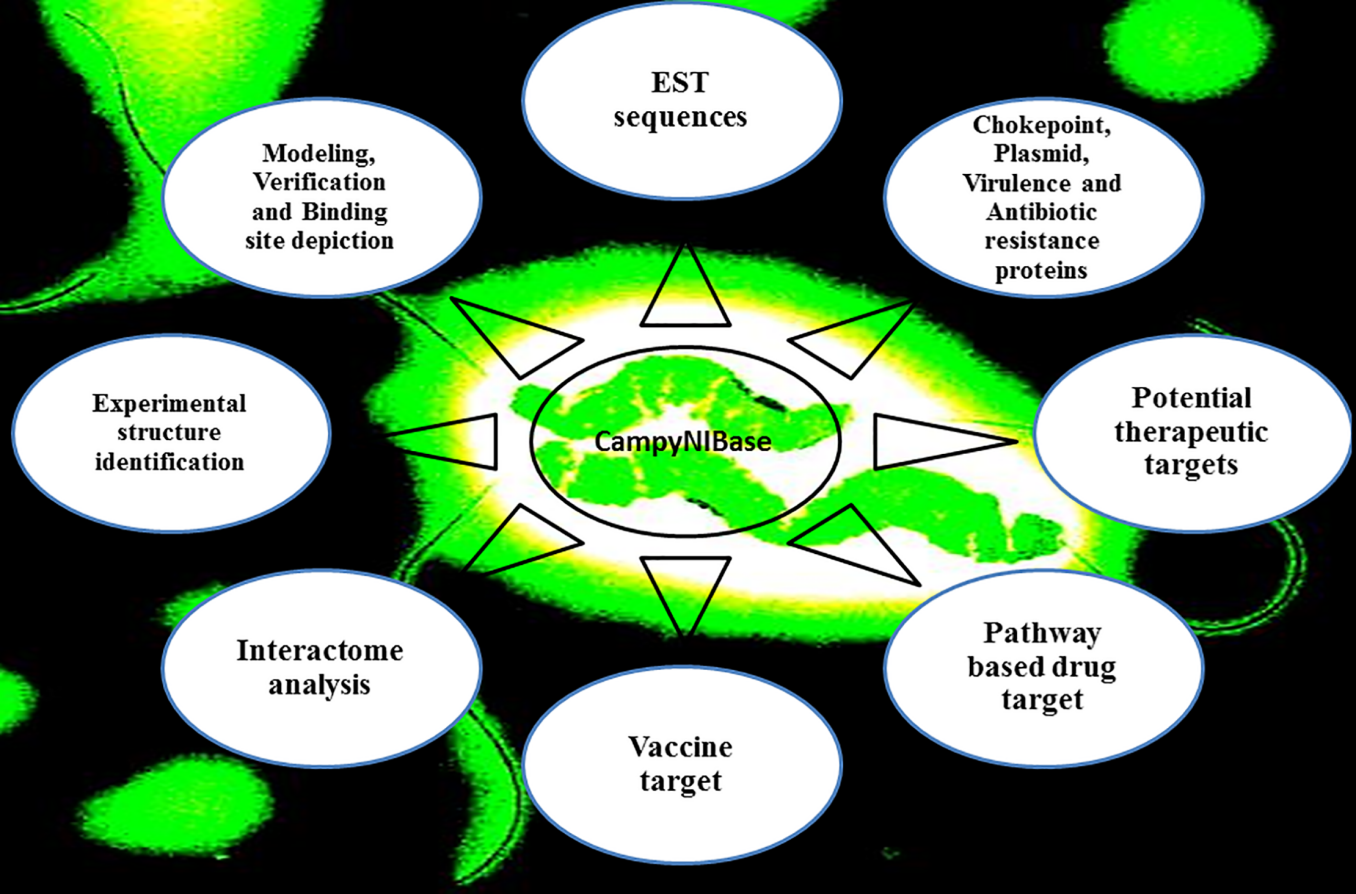
Architecture of ‘CampyNIBase’ database. The contents of developed database were categorized with different types of important relevant information.

**Fig 5:**
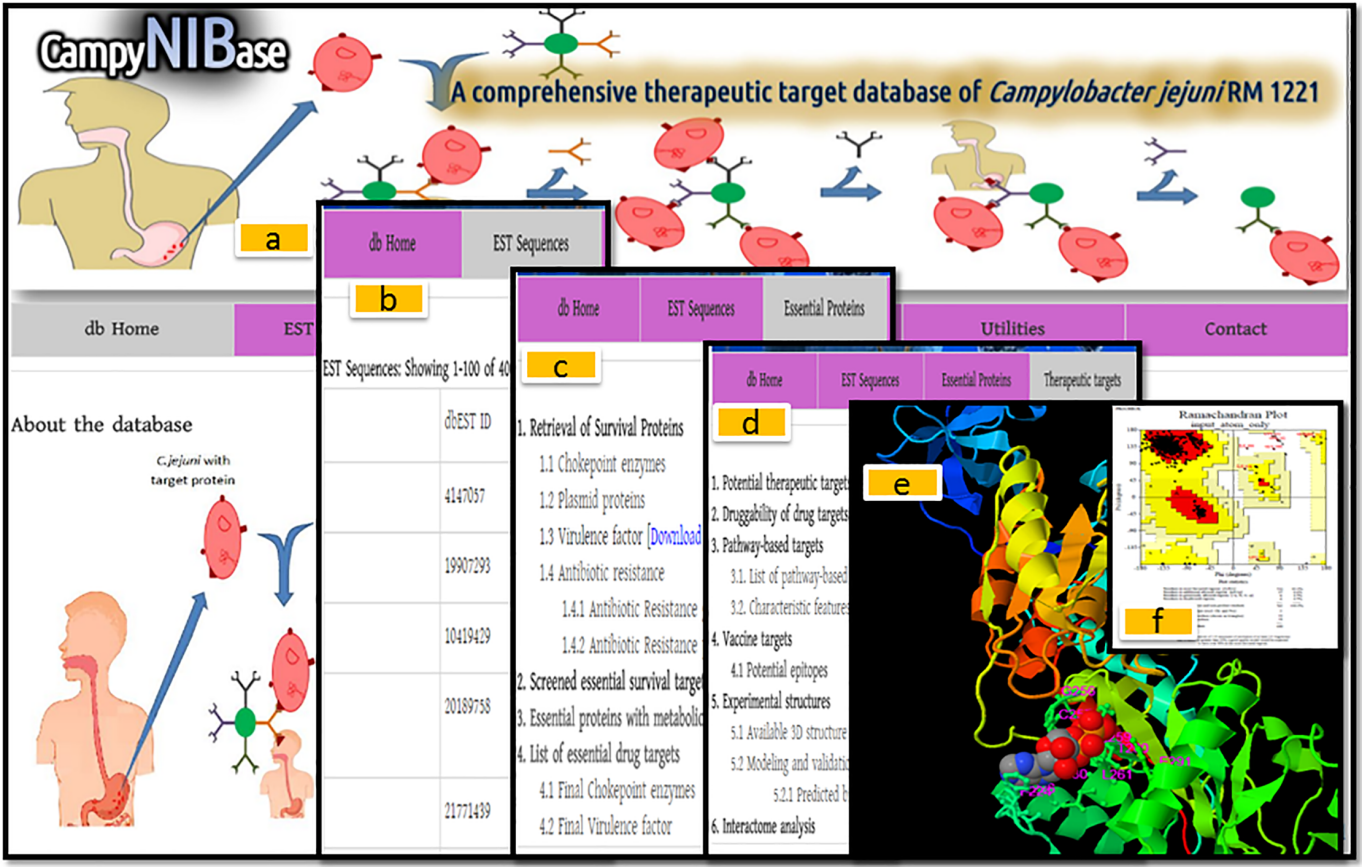
The snapshot of the ‘CampyNIBase’ database. (a) Homepage tab: summary of *campylobacter jejuni* RM1221. (b) EST tab: EST related information. (c) Essential proteins tab: essential proteins of *campylobacter jejuni* RM1221. (d) Therapeutic targets tab: identified targets and prioritization. (e) Information of built and experimental 3D structure. (f) Model verification.

We have deposited all the potential targets into our databases under the section of Essential proteins and Therapeutic targets. We have also stored the predicted EST sequence of *CjR* strain as the EST sequences are also important for denoting potential epitopes as well as vaccine and drug development (Figs 4 and 5).Furthermore, We have provided the link of our selected targets to be accessible to the other strains including *Campylobacter jejuni* subsp. doylei 269.97, *Campylobacter jejuni* subsp. jejuni 81–176, *Campylobacterjejuni* subsp. jejuni 81116 and *Campylobacter jejuni* subsp. jejuni NCTC 11168 directly via our database (Please refer to utility section of the database).

## Discussion

To our knowledge the current subtractive analysis performed in this study is the first report on computational analysis to identify and characterize therapeutic targets of *CjR*. We have selected *CjR* strainof campylobacter which is most prevalent in Bangladesh among the others. In molecular level, *CjR* strain is reported as one of the most frequent etiologic agent to cause about 65% diarrheal diseases in different clinical isolates in Bangladesh [74,75]. Besides this, technically to work with *CjR* strain in Bioinformatics analysis is advantageous asthis strain is well annotated with complete genome in KEGG and KAAS database and other bioinformatics resources as well. We have exploited total proteins of *CjR* for pathway based drug design. Whole proteome annotation identified a substantial number (2218) of protein sequences through various databases and literature searches. In this study, we have particularly focused on chokepoint enzymes, plasmid proteins, antibiotic resistance genes which are essential for *CjR*. In a metabolic reaction, only a specific substrate can be consumed and specific product produced during catalysis by a specific enzyme. This type of reaction is known as a chokepoint reaction and the enzyme that catalyzes the reaction is called a chokepoint enzyme. Therefore, certain chokepoint enzymes of a bacterial strain have potential to be a promising drug target [23]. As plasmid proteins can confer unique characteristics to a specific strain, we have collected their sequences through literature survey. In addition, antibiotic resistances by *C*. *jejuni* are mutations in certain genes. *C*. *jejuni* strains that have acquired multiple antibiotic resistances (MAR) overexpress *cmeB* compared to normal strains [28]. Many *C*. *jejuni* strains harbour point mutations in the *gyrA* gene to facilitate their resistance to fluoroquinolone [29]. Moreover, another resistance gene called *aphA-7*, which encodes a kanamycin resistant phosphotransferase, is found to be native in the genome of numerous *Campylobacter* species [30]. Usually, successful pathogenic bacteria rely on multiple virulent factors for survival and effective replication inside a host which in turn makes them potential drug targets (S1–S5 Tables).

The annotated proteome was filtered for duplicate and smaller protein sequences to avoid any ambiguity and identify ideal proteins respectively. As *CjR* is responsible for food poisoning and diarrhea, the genes responsible may be common to other bacterial species that also cause diarrhea and food poisoning. In addition, these genes can be important for their survival. To sort out these gene products/proteins, we have also filtered the proteins by subjecting them to BLASTp against the other bacterial species responsible for food poisoning, available in DEG 10 database [54]. Many of the successfully identified candidate drug targets revealed that they are involved in various metabolic pathways, essential for pathogen survival and absent in the host organism [76]. We have analyzed the filtered data for these properties and all the homologous human proteins were removed through BLASTp analysis. A further challenge when developing drugs against bacterial proteins is that humans contain a wide variety of bacterial species in their gut microbiome including symbiotic commensal bacteria. The proteins of these beneficial floras should ideally not be targeted. To avoid this problem BLASTp against these floras was also carried out. In this case, we found no protein that matched with the proteins of these floras.

KEGG database [55] contains genes of both complete and incomplete genomes. Genes of complete genomes are annotated by K numbers. For detailed analysis of proteins in KEGG database, annotation is required. To collect annotated proteins, KEGG Automatic Annotation Server (KAAS) [56] was used. Following annotation, we could easily identify proteins involved in pathways common to *CjR* and *H*. *sapiens*. These common proteins were removed to avoid cross targeting of human pathways by a pathway based drug. Subsequently, proteins belonging to unique pathways of *C*. *jejuni* were categorized based on the presence of proteins involved in either one or more than one pathway. Proteins found in one pathway were collected to aid in designing of pathway based drugs. Proteins with no involvement in pathway were not considered, as the aim of this study is to estimate pathway based drug targets. Again, proteins involved in more than one pathway were also excluded for the sake of simplicity of designing a pathway based drug. Some proteins were involved in only KEGG module. These are also not eligible as the module is a part of pathway, rather than whole pathway. These all ineligible proteins, other than pathway based drug targets, were named peptide drug targets. We have followed some published work in some reputed journal in which the most antigenic protein was selected for the peptide based epitope design [77–82]. A simple analysis was applied to them. The antigenicity of every peptide drug target was estimated by Vaxijen [59] as they might be helpful for vaccination. We have predicted T-cell and B-cell epitope from the highest probable antigenic target (S1 Fig, S16 and S17 Tables).

Thus, by this systematic subtraction analyses, owing to the considerations for all possible vital parameters, 38 potential therapeutic targets were identified (S6–S12 Tables). We have prioritized some pathway based identified therapeutic targets through characterization for certain properties such as subcellular localization, antigenicity, interactome, drug binding as well as whole pathway targeting ability. These analyses were handful for dividing them into drug or vaccine target. Comparing both subcellular location and antigenic score of all the 38 targets, N-acetylmuramoyl-L-alanine amidase, KEGG ID: CJE1405, was found as having the most potential as a vaccine target (S13–S20 Tables). According to DrugBank 3.0 [67] currently no approved drug or vaccine has been designed against this target and further wet lab validation experiments should be planned. Overall the characterization here revealed 12 drug targets based on interactome analysis, 5 targets to be dealt with FDA approved drugs and 1 for the pathway based drug target (Fig 3). In interacting genes analysis, it was found that a cytoplasmic protein, CJE0890, interacts with four genes and these interactions are common to all the *C*. *jejuni* strains available in STRING 10 (Table 3). So, it might become an essential target protein of *CjR* to design a drug against. Another important cytoplasmic protein, CJE0390, shows three interactions and there are available FDA approved drugs that target it (Table 3).

A drug target can be either a single protein or the whole pathway and drugs can be designed to target whole pathway. Pathways containing more eligible target proteins were considered as a target pathway with higher potential. From our analysis, the peptidoglycan biosynthesis pathway was found to contain the highest number of eligible target proteins and considered as the most significant target pathway.

As the *in silico* identified effective therapeutic [83–85], the probable vaccine and drug targets of *CjR* identified through this *in silico* approach are expected to contribute to development of new effective drugs and vaccines to treat campylobacteriosis. Our developed database (Figs 4 and 5) might also enable further exploration and characterization of *CjR* for the development of *CjR* specific therapeutics.

## Conclusions

The approach used in this study could corroborate to a powerful channel of analysis with rational accuracy toward identification of important essential genes in bacteria. This study identified one vaccine, 2 drug targets and 1 pathway based drug target. These potential therapeutic targets could be further validated experimentally through the drug and vaccine design pipelines. Together with 3D structures and other data, a comprehensive database ‘CampyNIBase’ has been developed to assist future *CjR* research.

## Supporting information

S1 Table Final chokepoint proteins. (XLSX)

S2 Table Plasmid protein. (XLSX)

S3 Table Final virulence factors. (XLSX)

S4 Table Final resistant protein. (XLSX)

S5 Table Combined total proteins. (XLSX)

S6 Table Proteins after duplicate rejection. (XLSX)

S7 Table Eligible protein collection. (XLSX)

S8 Table Selected filtered by BLASTp against HUMAN. (XLSX)

S9 Table Screened essential proteins by DEG. (XLSX)

S10 Table Proteins with KO. (XLSX)

S11 Table Final drug targets with pathway details. (XLSX)

S12 Table Final drug targets. (XLSX)

S13 Table Cellular localization. (XLSX)

S14 Table Antigenic prediction. (XLSX)

S15 Table Potential epitopes. (XLSX)

S16 Table T cell and MHC-I molecules. (XLSX)

S17 Table T-cell epitopes with epitope conservancy. (XLSX)

S18 Table Interactome analysis. (XLSX)

S19 Table Druggability. (XLSX)

S20 Table Pathway anlysis. (XLSX)

S1 Fig Prediction of B-cell antigenic properties. (A) Bepipred linear epitope prediction. (B) Chou and Fasman beta-turn prediction. (C) Emini surface accessibility prediction.(D) Karplus and Schulz flexibility prediction. (E) Kolaskar and Tongaonkar antigenicity prediction. (F) Parker hydrophilicity prediction. The x-axis and y-axis represent the sequence position and corresponding antigenic properties score, respectively. The threshold level was set as default parameter of the server. The regions were shown in yellow color above the threshold value to be predicted as B-cell epitope. All the cross referencing data showed peptide region NNEKENQKP (302–310) might be predicted as B-cell epitope.
(DOCX)
