# Supplementary material for: Pathway based therapeutic targets identification and development of an interactive database CampyNIBase of Campylobacter jejuni RM1221 through non-redundant protein dataset
Source: PLoS One. 2018 Jun 8;13(6):e0198170. doi: 10.1371/journal.pone.0198170 (PMC5993290; doi:10.1371/journal.pone.0198170)
Supplement: S1 Fig — (A) Bepipred linear epitope prediction. (B) Chou and Fasman beta-turn prediction. (C) Emini surface accessibility prediction.(D) Karplus and Schulz flexibility prediction. (E) Kolaskar and Tongaonkar antigenicity prediction. (F) Parker hydrophilicity prediction. The x-axis and y-axis represent the sequence position and corresponding antigenic properties score, respectively. The threshold level was set as default parameter of the server. The regions were shown in yellow color above the threshold value to be predicted as B-cell epitope. All the cross referencing data showed peptide region NNEKENQKP (302–310) might be predicted as B-cell epitope. (DOCX) [file pone.0198170.s021.docx]

**
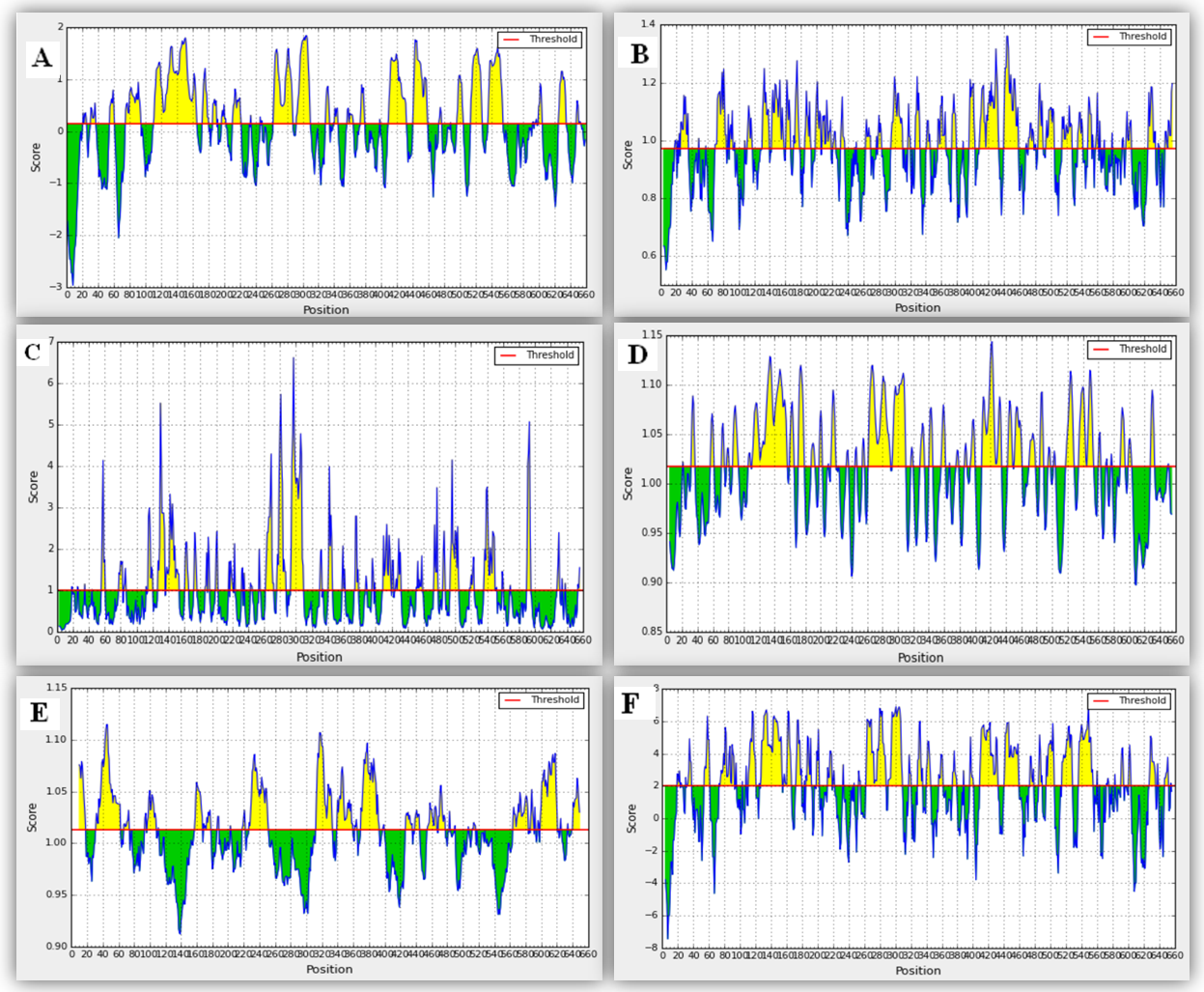
**

**S1 Fig. Prediction of B-cell antigenic properties** (A) Bepipred linear epitope prediction. (B) Chou and Fasman beta-turn prediction. (C) Emini surface accessibility prediction. (D) Karplus and Schulz flexibility prediction. (E) Kolaskar and Tongaonkar antigenicity prediction.  (F) Parker hydrophilicity prediction. The x-axis and y-axis represent the sequence position and corresponding antigenic properties score, respectively. The threshold level was set as default parameter of the server. The regions were shown in yellow color above the threshold value to be predicted as B-cell epitope. All the cross referencing data showed peptide region NNEKENQKP (302-310) might be predicted as B-cell epitope.
